# Treatment outcomes using CyberKnife for brain metastases from lung cancer

**DOI:** 10.1093/jrr/rru092

**Published:** 2014-10-25

**Authors:** Keisuke Tamari, Osamu Suzuki, Naoya Hashimoto, Naoki Kagawa, Masateru Fujiwara, Iori Sumida, Yuji Seo, Fumiaki Isohashi, Yasuo Yoshioka, Toshiki Yoshimine, Kazuhiko Ogawa

**Affiliations:** 1 Department of Radiation Oncology, Osaka University, 2-2 (D10) Yamadaoka, Suita, Osaka 565-0871, Japan; 2 Department of Neurosurgery, Osaka University, 2-2 (D10) Yamadaoka, Suita, Osaka 565-0871, Japan

**Keywords:** stereotactic radiosurgery, CyberKnife, brain metastases, lung cancer

## Abstract

We investigated the clinical outcomes following treatment using stereotactic radiosurgery (SRS) and fractionated stereotactic radiotherapy (SRT) for brain metastases from lung cancer. A total of 67 patients with 109 brain metastases from lung cancer treated using CyberKnife between 1998 and 2011 were retrospectively analyzed. SRS (median dose, 24 Gy) was used to treat 79 lesions, and 3-fraction SRT (median dose, 30 Gy) was used to treat 30 lesions. The median follow-up time was 9.4 months (range, 0.4–125 months). The 1-year local control rate was 83.3%, and the 1-year distant brain failure rate was 30.1%. The median survival time was 13.1 months, and the 1- and 3-year overall survival (OS) rates were 54.8% and 25.9%, respectively. On multivariate analysis, three factors were found to be statistically significant predictors of OS: (i) presence of uncontrolled primary disease [hazard ratio (HR) = 3.04; *P* = 0.002]; (ii) Brinkman index (BI) ≥ 1000 (HR = 2.75; *P* = 0.007); and (iii) pulmonary metastases (HR = 3.54; *P* = 0.009). Radionecrosis and worsening of neurocognitive function after radiosurgery were observed in 5 (7%) and 3 (4%) patients, respectively. Our results indicated that SRS/SRT for brain metastases from lung cancer was effective. Uncontrolled primary disease, high BI, and pulmonary metastases at treatment were significant risk factors for OS.

## INTRODUCTION

Metastatic brain tumors are the most common intracranial neoplasms in adults and are a significant cause of morbidity and mortality. The overall incidence rate of brain metastases from various primary sites is 9.6%. In particular, brain metastases from lung cancer have the highest incidence rate of 19.9% [1]. Recently, brain metastases have been more frequently diagnosed because of improvement in the detection of small metastases by magnetic resonance imaging (MRI) and longer survival rates because of improved systemic therapies, including molecular targeting therapy.

The clinical presentations of cerebral metastases are variable according to the location of the lesions. Hemiparesis, altered mental status, gait ataxia, and hemisensory loss are common signs [2]. Because these symptoms significantly impair the quality of life (QOL) of patients, the treatment of brain metastases is important.

Treatment options for patients with brain metastases include medical management, surgical resection, whole brain radiotherapy (WBRT), stereotactic radiosurgery (SRS) and fractionated stereotactic radiotherapy (SRT). However, there is a lack of consensus on the optimum treatment strategy. Although WBRT is a routine treatment for patients with brain metastases, late complications such as neurocognitive disorders can impair QOL of patients [3–5]. Moreover, the concept of oligometastases has emerged in brain metastasis. If the number of brain metastases is limited, WBRT can be replaced by surgical resection and SRS [6]. Tsao *et al*. performed a meta-analysis of patients with newly diagnosed brain metastases (1–4 tumors) and concluded that SRS alone should be considered as a routine treatment option rather than WBRT plus SRS to achieve favorable neurocognitive outcomes [7]. Moreover, Yamamoto *et al*. recently conducted a prospective study and suggested that SRS in patients with 5–10 brain metastases is not inferior to that in patients with 2–4 brain metastases [8]. SRS has increasingly had a significant role in management of brain metastases as an initial treatment.

In the RTOG 90–05 trial, the maximum tolerated doses of SRS were defined and unacceptable toxicity was more likely in patients with larger metastases [9]. SRT would theoretically improve the sparing of critical structures, and consequently could limit the long-term side effects of normal tissues, presumably with a low α/β ratio. In this sense, SRT could have a primary role in the treatment of brain metastases, especially when these are close to critical structures or are large. SRS/SRT is performed with Gamma Knife or linear accelerator-based stereotactic machines such as CyberKnife, which is an established modern noninvasive technology for intracranial and extracranial radiosurgery. Gamma Knife had provided single fraction radiosurgery but needed the use of an invasive skull pin fixation frame system. Recently, however, fractionated Gamma Knife radiotherapy using a non-invasive frame system with submillimeter accuracy has emerged and been used for patients with intracranial tumors [10]. Although many studies investigating outcomes of SRS have been published, few have investigated outcomes of SRS/SRT for the management of brain metastases from lung cancer. In this study, we retrospectively analyzed treatment outcomes for patients with brain metastases from lung cancer treated using CyberKnife SRS/SRT in our institution.

## MATERIALS AND METHODS

### Patient characteristics

This retrospective study was approved by the institutional review board of Osaka University Medical Hospital. A total of 67 patients with brain metastases from lung cancer were treated with SRS/SRT using CyberKnife between 1998 and 2011. All the patients participating in this study submitted informed consent prior to treatment. Pretreatment evaluation included complete medical history, physical examination, complete blood cell count, biochemical screening profile, chest radiography, thoracic computed tomography (CT), and brain MRI. Clinical tumor, node and metastases staging were defined according to the seventh edition of the Union for International Cancer Control – American Joint Committee on Cancer staging system.

The patient characteristics are listed in Table 1. The median patient age was 63 years (range, 29–82 years). The sex distribution was 46 males (69%) and 21 females (31%). Of the 67 patients, 64 (96%) were previously diagnosed with non-small-cell lung cancer (NSCLC) and 3 (4%) were diagnosed with small-cell lung cancer (SCLC). In the NSCLC patient group, 53 patients (79%) were diagnosed with adenocarcinoma, eight patients (12%) with squamous-cell carcinoma, two patients (3%) with large-cell carcinoma, and one patient (1.5%) as ‘not otherwise specified’. The performance status (PS) according to the Eastern Cooperative Oncology Group criteria was 0–1 in 47 patients (70%) and 2–3 in 20 patients (30%). The median Brinkman index (BI) was 450 (range, 0–2580). The median volume of the metastatic lesions was 1.2 cm^3^ (range, 0.01–26.8 cm^3^), and the number of brain lesions was one in 37 patients (55%), two in 19 patients (28%), three in seven patients (10.5%), and ≥ 4 in four patients (6%). Primary disease control was achieved for 37 patients (55%), and 34 patients (51%) had extracranial metastases at the time of CyberKnife treatment. Prior WBRT (at 30 Gy in 10 fractions) was administered to 11 patients (16%). In principle, WBRT is used for patients with ≥4 brain metastases in our institution.

**Table 1.** Patient characteristics

Characteristic		Characteristic	
Age (years)	median 63 (29–82)	Uncontrolled primary disease	42% (28/67)
Sex		Extracranial metastasis	51% (34/67)
Male	69% (46/67)	Lung	21% (14/67)
Female	31% (21/67)	Bone	27% (18/67)
Histology of primary tumor		Liver	9% (6/67)
NSCLC	96% (64/67)	Prior WBRT	16% (11/67)
SCLC	4% (3/67)	CyberKnife dose (BED_10_)	
Performance status (ECOG)		Radiosurgery	
0–1	70% (47/67)	20 Gy (60)	5% (5/109)
2–3	30% (20/67)	22 Gy (70.4)	1% (1/109)
Brinkman index	median 450 (0–2580)	24 Gy (81.6)	23% (25/109)
Neurological dysfunction	40% (27/67)	25 Gy (87.5)	44% (48/109)
Neurocognitive disorder	12% (8/67)	Fractionated radiotherapy	
Tumor volume (cm^3^)	median 1.2 cm^3^ (0.01–26.8)	18 Gy/3 Fr (28.8)	1% (1/109)
Number of brain metastasis		24 Gy/3 Fr (43.2)	1% (1/109)
1	55% (37/67)	27 Gy/3 Fr (51.3)	2% (2/109)
2	28% (19/67)	30 Gy/3 Fr (60)	20% (22/109)
3	10% (7/67)	33 Gy/3 Fr (69.3)	2% (2/109)
≥4	6% (4/67)	36 Gy/3 Fr (79.2)	2% (2/109)

NSCLC = non-small-cell lung cancer, SCLC = small-cell lung cancer, WBRT = whole brain radiotherapy, BED_10_ = biological equivalent dose for α/β = 10, Fr = fraction.

### Treatment

All the patients underwent SRS/SRT using CyberKnife (Accuray Inc., Sunnyvale, CA, USA). CyberKnife is equipped with a 6-MV linear accelerator mounted on a computer-controlled robotic arm. During treatment, all patients were in the supine position and fitted with a thermoplastic mask for immobilization. CT images of 1-mm slice thickness were fused with contrast-enhanced MRI. The clinical target volume (CTV) was defined as the enhanced lesion observed by contrast-enhanced MRI. The planning target volume (PTV) was generated by adding a margin of 1 mm to the CTV. The organs at risk, including the eyes, lenses, optic nerves, optic chiasm, brainstem and spinal cord, were contoured. Plans were generated using the Multiplan inverse treatment-planning algorithm (Accuray Inc.).

SRS with a median dose of 24 Gy (range, 20–25 Gy) prescribed to D90 (the radiation dose received by 90% of the PTV) was used to treat 79 lesions. Three-fraction SRT with a median dose of 30 Gy (range, 18–36 Gy) prescribed to D90 was used to treat 30 lesions. The tumor volume was significantly greater in SRT (median, 6.1 cm^3^; range, 3.4–26.8 cm^3^) than in SRS (median, 0.9 cm^3^; range, 0.01–8.6 cm^3^) with a probability (*P*) value < 0.001 (2-sided *t*-test).

Target displacements caused by patient movements during treatment were automatically corrected. Stereoscopic X-ray images acquired during treatment were coregistered with a set of digitally reconstructed radiographs (DRRs) from the dose-planning CT. A displacement vector was calculated by matching pairs of stereoscopic live images with DRRs. CyberKnife has submillimeter accuracy [11].

### Evaluation of clinical outcomes

In principle, patients underwent contrast-enhanced MRI after 4 weeks and every 3 months thereafter. The last visit or date of contact was used to censor surviving patients at the time of analysis. The median follow-up time was 9.4 months (range, 0.4–125 months).

We evaluated tumor response using the Response Evaluation Criteria in Solid Tumors (ver. 1.1) [12]. Complete response, partial response, and stable disease were categorized as local control (LC), and progressive disease was categorized as local failure. In addition, we evaluated distant brain failure (DBF), overall survival (OS), and adverse effects such as neurocognitive disorders and radionecrosis. We defined neurocognitive disorders as neurocognitive decline from a previous level of performance in complex attention, executive function, learning, memory or language, which was clearly written in the follow-up records by neurosurgeons and radiation oncologists if the records had no quantitative evaluation of neurocognitive function.

### Statistical analysis

Data analysis was performed using JMP pro 10 statistical software (SAS Institute Inc., Cary, NC, USA). LC, DBF and OS rates were calculated using the Kaplan–Meier method. The log-rank test was used for univariate analysis to assess predictive factors associated with OS and LC. In addition, the Cox proportional hazard model was used for multivariate analysis. Estimated hazard ratios (HRs) were calculated. A *P* value <0.05 was considered statistically significant. Statistical tests were based on a 2-sided significance level.

The following clinical factors were investigated for their association with OS: age, gender, histology of primary tumor, PS, BI, neurological dysfunction, neurocognitive disorder, tumor volume, number of metastases, primary disease control, extracranial metastasis, prior WBRT, LC and DBF. In addition, we performed univariate analysis of age, gender, histology of primary tumor, PS, BI, tumor volume, primary disease control, prior WBRT, prescription dose [calculated as the biological equivalent dose for α/β = 10 (BED_10_)], and fractionation for LC.

## RESULTS

### LC and DBF

**Fig. 1.**
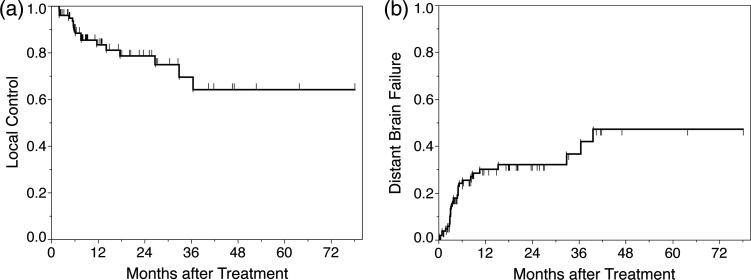
Local control and distant brain failure.

The 1- and 2-year LC rates were 83.3% and 78.5%, respectively (Fig. 1). Of the 13 patients with local failure, four received WBRT, two underwent neurosurgery and postoperative WBRT, two were treated using CyberKnife, and one received systemic therapy. On univariate analysis, we identified that the age of ≥70, the male sex, a BI of ≥1000, prior WBRT, a tumor volume of ≥18 cm^3^, and a prescription dose (BED_10_) of ≤ 60 Gy were risk factors for LC. However, fractionation was not a risk factor for LC (Table 2). We could not perform multivariate analysis because of the small sample size.

**Table 2.** Results of univariate analysis of risk factors associated with local control

Characteristic		Univariate *P* value
Age	≥70	0.001
Sex	male	0.030
Performance status	≥2	0.102
Brinkman index	≥1000	0.003
Histology	AC	0.053
Tumor volume	≥18 cm^3^	0.020
Prescription dose (BED_10_)	≤60 Gy	0.023
Fractionation	SRT	0.596
Prior WBRT	Yes	0.015
Controlled primary disease	No	0.252

AC = adenocarcinoma, BED_10_ = biological equivalent dose for α/β = 10, WBRT = whole brain radiotherapy.

The 1- and 2-year DBF rates were 30.1% and 32.1%, respectively (Fig. 1). Of the 22 patients with DBF, nine were treated using CyberKnife, seven received WBRT, two underwent Gamma Knife SRS, one received systemic therapy, and the records of four patients had no clinical information regarding further treatment.

### OS

**Fig. 2.**
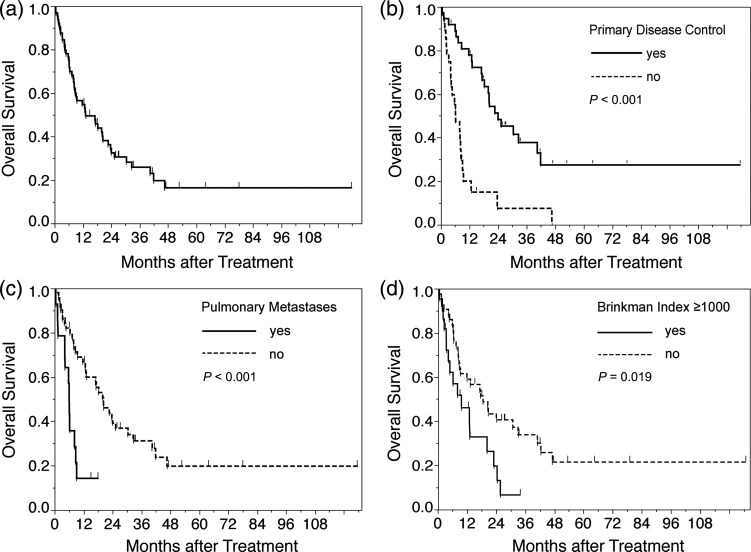
Overall survival. (**a**) Overall survival and Kaplan–Meier survival curves for prognostic factors. (**b**) Survival curves by primary disease control. (**c**) Survival curves by pulmonary metastases. (**d**) Survival curves by Brinkman index.

The median survival time (MST) was 13.1 months, and the 1- and 3-year OS rates were 54.8% and 25.9%, respectively (Fig. 2). As shown in Table 3, three factors were found to be statistically significant predictors of OS on multivariate analysis: (i) presence of uncontrolled primary disease at the time of treatment using CyberKnife (HR = 3.04; *P* = 0.002); (ii) BI ≥ 1000 (HR = 2.75; *P* = 0.009); and (iii) pulmonary metastases (HR = 3.54; *P* = 0.007). The MST for patients with controlled primary disease was 24 months and that for patients with uncontrolled primary disease was 6.2 months. The MST for patients with BI ≥ 1000 and BI < 1000 was 9.4 and 18.3 months, respectively. The MST for patients with and without pulmonary metastases was 6 months and 20.2 months, respectively. Interestingly, LC and DBF were not predictive factors for OS.

**Table 3.** Results of univariate and multivariate analysis of factors associated with overall survival

Characteristic		Univariate *P* value	Multivariate *P* value	Multivariate Hazard Ratio
Age	≥70	0.856		
Sex	male	0.013	0.214	1.54 (95% CI; 0.78–3.30)
Performance status	≥2	0.994		
Brinkman index	≥1000	0.019	0.007	2.75 (95% CI; 1.32–5.77)
Neurological dysfunction		0.488		
Neurocognitive disorder		0.775		
Histology	AC	0.098		
Number of brain metastases	≥3	0.115		
Prior WBRT		0.098		
Pulmonary metastases		<0.001	0.009	3.54 (95% CI; 1.39–8.58)
Liver metastases		0.008	0.619	1.28 (95% CI; 0.45–3.18)
Bone metastases		0.061		
Adrenal metastases		0.394		
Uncontrolled primary disease		<0.001	0.002	3.04 (95% CI; 1.54–6.03)
LC		0.163		
DBF		0.373		

AC = adenocarcinoma, WBRT = whole brain radiotherapy, CI = confidence interval, LC = local control, DBF = distant brain failure.

### Complications

Radionecrosis was observed in five patients (7%). The median time from treatment using CyberKnife to the diagnosis of radionecrosis was 10.3 months (range, 9–21.1 months), and the 2-year incidence rate of radionecrosis was 12.9%. None of the patients who developed radionecrosis were symptomatic, and none required medical treatment such as steroids. In addition, neurocognitive disorders were observed in three patients (4%), and the 2-year incidence rate of neurocognitive disorders was 7.4%. Other complications included convulsion in two patients (2.9%), intratumoral hemorrhage in one patient (1.5%), and brain edema in one patient (1.5%).

## DISCUSSION

SRS/SRT is increasingly used for the initial treatment of a limited number of patients with brain metastases. However, the use of SRS/SRT for the treatment of brain metastases from lung cancer has not been well investigated. As shown in Table 4, we found only seven published series on non-CyberKnife SRS/SRT for brain metastases from lung cancer. LC was achieved in 77–98%, and the MST was 7–14.5 months. Predictive factors for poor OS varied in these series, such as male gender, poor PS, active extracranial metastases, uncontrolled primary disease, histology of adenocarcinoma, a relatively long period from original diagnosis of lung cancer to brain metastases, large number of metastatic lesions, and higher recursive partitioning analysis class [13–19]. Our treatment results (1-year LC rate of 83.3% and MST of 13.1 months) are in accordance with those of previous studies. Interestingly, we found that a greater BI and pulmonary metastases are new predictive factors for poor OS. We suspected that the use of gefitinib was associated with a better OS. However, on univariate analysis, we found that gefitinib was not a significant predictive factor for OS (data not shown). This may be because only seven patients were treated with gefitinib, whereas most patients were treated prior to the widespread use of EGFR inhibitors. Recently, the smoking status in patients with lung cancer has been shown to be a prognostic factor for OS. Tsao *et al*. performed retrospective analysis of 873 patients with NSCLC (Stage III–IV) who were treated with frontline chemotherapy. Never smokers (*n* = 137) had higher response rates than former or current smokers (19% versus 8% versus 12%, respectively; *P* = 0.004), and improved OS (*P* < 0.0001). Never-smoking status remained an independent predictor in multivariate analysis including adjustment for age, gender, stage, and performance status, with an HR of 1.47 for former smokers (*P* = 0.003) and an HR of 1.55 for current smokers (*P* = 0.0004) [20]. Kawaguchi *et al*. also retrospectively analyzed 1499 never-smokers and 3455 ever-smokers with advanced Stage IIIB and IV lung cancer who had received cytotoxic chemotherapy. The smoking status was a significant prognostic factor (never-smoker versus ever-smoker; HR = 0.880, *P* = 0.0105) [21]. Although these studies suggest that a higher BI could be a risk factor for OS in our study, further investigations are required to evaluate smoking status in patients with brain metastases as a predictive factor for OS. The DBF rate of 30.4% at 1 year was consistent with the results of a recently published series, and DBF does not affect OS or the incidence of neurological death [22–25]. Recursive partitioning analysis class, which is a well-known prognosis factor, was not evaluated because we did not routinely evaluate Karnofsky performance status.

**Table 4.** Summaries of published series about treatment outcomes of stereotactic radiosurgery for brain metastases from lung cancer

Author	Histology (*n*)	Modality	Median survival time	Local control
Serizawa *et al*. [13]	SCLC (34) NSCLC (211)	GK	SCLC: 9.1 months NSCLC: 8.6 months	SCLC: 94.5%, NSCLC: 98% at 1 year
Sheehan *et al.* [14]	NSCLC (273)	GK	7 months	86%
Gerosa *et al*. [15]	SCLC (33) NSCLC (471)	GK	14.5 months	94% at 1 year
Mariya *et al*. [16]	NSCLC (84)	Linac	9 months	77% at 1 year
Motta *et al*. [17]	NSCLC (373)	GK	14.2 months	NR
Wegner *et al*. [18]	SCLC (44)	GK	9 months	86% at 1 year
Ma *et al*. [19]	NSCLC (171)	Linac	SRT + WBRT: 13 months, SRT alone: 9 months	83.3% at 1 year
Present study	SCLC (3) NSCLC (64)	CK	SRS/SRT: 13.1 months	83.3% at 1 year

SCLC = small-cell lung cancer, NSCLC = non-small-cell lung cancer, GK = Gamma Knife, NR = not reported, SRS = stereotactic radiosurgery, WBRT = whole brain radiotherapy, CK = CyberKnife.

Univariate analysis identified that age ≥ 70, the male sex, a BI of ≥ 1000, prior WBRT, a tumor volume ≥ 18 cm^3^, and a radiation dose (BED_10_) of ≤ 60 Gy were significant risk factors for LC. Because of the small sample size, we could not perform multivariate analysis. Lee *et al*. retrospectively analyzed 109 patients with 119 large brain metastases [median tumor volume of 16.8 cm^3^ (6.0–74.8 cm^3^)] treated with Gamma Knife, and identified prior WBRT as a risk factor for local failure [26]. Yang *et al*. also performed retrospective analysis of 70 patients with a brain metastasis (median tumor volume of 14 cm^3^ (6.0–32 cm^3^)), and identified prior WBRT and tumor volume >16 cm^3^ as risk factors for local failure [27]. Therefore, a higher prescription dose may be required in patients with larger metastases or who have undergone prior WBRT. Further investigation is required to clarify prognostic factors for LC.

Adverse effects, including neurocognitive disorders and radionecrosis, were identified in our analysis. According to previous reports, the rate of postradiosurgical neurocognitive disorders is 24–48.1% [3, 4]. In our analysis, neurocognitive disorders were identified in 4% of patients. However, we may have underestimated the occurrence of neurocognitive disorders because we did not routinely evaluate neurocognitive function using a quantitative method such as the mini-mental state examination. We performed a literature search and retrieved six published series regarding postradiosurgical radionecrosis. After Gamma Knife SRS, 24–38.4% of patients developed radionecrosis. Significant risk factors for radionecrosis were reported as brain V10 and V12 [28–32]. Recently, Inoue *et al*. performed dosimetric analysis to identify complications after SRT using CyberKnife and found that radionecrosis occurred in 6.2% of patients and that brain V14 (single dose equivalence) ≥7 cm^3^ was a risk factor for radionecrosis [33]. In our analysis, radionecrosis was found in 7% of patients. We think that our series might be at lower risk for radionecrosis, possibly because the PTV volume of our series tended to be smaller than that of other published series, or possibly because we treated larger metastases using SRT.

With regard to the shortcomings of this study, we were unable to perform dose–volume histogram analysis because of the retrospective nature of this study, which made it impossible to analyze old dosimetric parameters. Therefore, further investigations are required to establish adequate dose restrictions to prevent radionecrosis following treatment using CyberKnife, particularly when employing a fractionated schedule.

In conclusion, our results showed the efficacy of using CyberKnife SRS/SRT to treat brain metastases from lung cancer. Uncontrolled primary disease, high BI, and pulmonary metastases are significant risk factors that affect OS. These findings should be useful for clinical practitioners who treat brain metastases using SRS/SRT.

FUNDING

Funding to pay the Open Access publication charges for this article was provided by the Japan Society for the Promotion of Science (JSPS) KAKENHI Grant No. 25861098.
